# Varicose Veins and Ulcers of the Legs

**Published:** 1838

**Authors:** 


### Art. X.—Varicose Veins and Ulcere oe the Legs.

Varicose veins of the legs are so frequent, that almost every physician is called upon to treat them. When complicated with ulcers their cure is important; and when no ulcers are present, the *alarm* they give to some patients, requires that we should be thoroughly acquainted with the results of the experience of those who have had ample opportunities. Among these, scarcely any European surgeon, would be of more authority with our readers, than Mr. Brodie of London. We shall therefore transfer to our pages the curative part of one of his lectures, in St. George's Hospital, as reported in the Medical Gazette for November last.

D.

"In those cases in which, from long neglect of varicose veins, the skin of the leg becomes red and irritable, you will be able to render the patient no service so long as he is going about, standing and walking as usual. The first thing to be done is, to confine him to his bed, or, at all events, to a sofa; but the safest method is to confine him to his bed, and the horizontal posture, so that the blood may not have to rise up in the leg against its own gravity. In many cases, nothing more is necessary than this; but in some instances, this will afford but very slow relief, and in all cases you may hasten the patient's recovery by adopting other method's in addition. I have frequently, in these cases, bled the patient in the vena saphena major, in the lower part of the thigh, near the inner condyle; and it is astonishing what relief that gives. It is not worth while to adopt this practice in all cases, but where you find the patient suffering more than usual from the inflamed state of the skin, you may very properly have recourse to it.

Bleeding in the vena saphena major is performed very easily in persons who are not very fat: place a bandage round the lower part of the thigh, let the patient put his leg into a pail of warm, water, and what with the warm water below and the bandage above, the vena saphena swells; you then open it with a lancet, and take away any quantity of blood you please. But, in a very fat person, bleeding from the vena saphena is not very easy to be accomplished, and as a substitute for it you may apply leeches to the inside of the thigh, or you may apply them in this situation in other cases, where you do not think that actual bleeding in the vena saphena is required. And here I must call to your recollection what I said respecting the application of leeches, under these circumstances, in my last lecture. Never apply leeches to the inflamed part, but always at some distance above it. If the whole skin of the leg be inflamed, then apply them on the inside of the thigh; if the leg be inflamed in the lower part and not in the upper, then apply them in the leg, but above the inflammation. Besides the application of leeches, you may, in the first instance, apply a rag, wetted with cold spirituous or saturnine lotion. When the inflammation of the skin has subsided, you may begin the use of bandages in the way which I described in the last lecture.

"In some cases, as I formerly told you, the skin is not only inflamed, but more or less excoriated, the cuticle being abraded to a greater or less extent, while the surface of the cutis secretes an ichorous fluid. Here, also, you may take away blood from the vena saphena major, or or from the inside of the thigh by leeches, and the patient will also derive benefit in these cases from the application of a saturnine lotion, though, for the most part, some mild cerate answers the purpose better. The zinc ointment or calamine cerate answers very well; but we use, in the hospital, a preparation known with us by the name of compound chalk ointment, which is much preferable. It is, if I am not mistaken, now introduced into the Pharmacopœia, under the name of ung. plumbi compositum. It is an excellent application in these and other cases, where the surfacc of the cutis is deprived of the cuticle. This ointment was invented by Dr. Kirkland, a celebrated practitioner, many years ago, in Leicestershire, and I believe it was commonly known under the name of Kirkland's neutral cerate. It is composed of diachylon plaster, olive oil, chalk, and distilled vinegar. How it should have ever entered into any man's head to make such a composition as this, I do not know; but the composition having been invented, I must say, it is a very useful one. The ointment should be spread on a linen rag, and applied in stripes round the leg, each stripe over-lapping the one below. In some cases, in addition to the use of chalk ointment, you will find advantage from washing the surface with a weak solution of nitrate of silver, in the proportion of two or three grains to an ounce of rose water. A strong solution would here be improper, but a weak solution is very useful.

I told you that in some cases there was œdema, a swelling of the leg and foot, in consequence of the inflammation of the cellular membrane, causing it to be infiltrated with coagulated lymph and serum. The treatment that is required under these circumstances is very nearly the same as that which is necessary where there is the inflammation of which I have just spoken. The patient should be kept in the horizontal posture; blood may be taken either from the vena saphena major, or by leeches from the thigh, and generally you will find the latter quite sufficient. You may apply a cold lotion in the first instance, but very soon, in these cases, you should begin to apply a bandage, such as will give a uniform support to the leg, from the toes to the knee.

In cases of varicose ulcers of the leg, if you find that the patient has neglected himself, that the ulcer is in a state of inflammation, foul and painful, as it often is, and the surrounding skin being in a state of inflammation also, you must keep the patient in bed, and treat him as if the leg were inflamed without the existence of the ulcer. But as soon as the inflammation of the ulcer and the surrounding parts has been relieved, you may begin the application of pressure. The pressure of a common roller will do a great deal of good, and formerly nothing else was recommended. But we find, now, that in cases of varicose ulcer, as in cases of indolent ulcer of the leg, you may very much assist the common roller by the addition of other means. One very good way of making pressure on a varicose ulcer, is to interpose between it and the bandage a piece of sheet lead, such as is used in anatomical museums for covering preparations. The lead should be made quite smooth, and larger than the ulcer, extending some way beyond its margin. This makes a very uniform pressure, and really does very well. But for the most part, we are in the habit of using pressure by means of plaster applied in a circular manner round the limb. It is common to employ stripes of linen, spread with soap or adhesive plaster; but I own that I very much prefer diachylon plaster; for both soap plaster and adhesive plaster will frequently irritate the skin, and bring on inflammation and pustules, but diachylon plaster scarcely ever produces this effect.

You have an opportunity of seeing stripes of diachylon plaster applied every day, and over and over again every day, in the wards of the hospital; and, therefore, it might seem almost superfluous for me to make any observations on the mode of applying them. But I find that new dressers very seldom apply them in the manner that I believe to be proper, and therefore I thall offer to you some observations on that subject.

In the first place, the stripes should be applied round the limb, the two ends crossing each other in front, the application beginning below every part has a double piece of plaster over it. Each of the stripes ought to overlap the one below by one half of its diameter. Thus every part has a double piece of plaster over it, and you secure more equal pressure than you could otherwise obtain. It is of great consequence that the plaster should be tight enough to give comfortable support to the limb, and at the same time not so tight as to make the limb swell below; for if it does produce this effect, it is very likely that it will bring on a sloughing of the sore. The plasters ought to make uniform pressure—that is, the pressure should be equal throughout; or if there be any difference in the degree of pressure, it ought to be greater below than above. If you do not attend to this point, the plaster above operates as a tight garter, and makes the parts below swell.

When you apply the plaster, it should always be with the heel raised, the patient lying flat on his back, so that the vessels of the leg may be emptied of their blood. The same plan should be adopted when the plaster is taken off. If the leg be hanging down at the time the plaster is applied, the veins are full of blood, and the plaster becomes too loose as soon as the patient puts his leg up.

The plaster, if there be much discharge, should be changed daily; but as the discharge becomes less in quantity, it may be changed every other day, or once in three days, and in some cases it may be left on even longer than that.

Frequently, in cases of varicose ulcer, you find the veins on each side of the leg just above the heel, and behind the ankles, formed into a varicose cluster. A bandage applied in the common manner does not sufficiently support these veins. The ulcer may be above, and you may cover it with a bandage; but if there be such veins as I have mentioned, below, you must not, for obvious reasons, leave them uncovered.

In order to support these viens, some stripes of plaster should be applied round the lower part of the heel, extending upwards, in a longitudinal direction, on each side of the leg. Let these be held firmly on while you apply the circular stripes over them, in order to keep them in their place. In this case also, in the application of the bandage, you ought to pursue the same course: a longitudinal bandage, extending under the heel and up each side of the leg, should be applied first, and this covered by a circular bandage afterwards. These may appear matters of little importance, but a great deal of your success in practice will depend on attention to such minutiæ. It is not enough to understand the case, to make a good diagnosis, and to know what remedies are to be employed; you should also take pains to apply these remedies in the best possible manner, otherwise they may fail in producing their effect. In some cases of varicose ulcer, you will promote the healing of the ulcer by touching it, every other day, with a strong solution of nitrate of silver in water, beginning with five or six grains to an ounce, and increasing the strength gradually. But I do not advise you, as a general rule, to put any application in the way of dressing under the plaster. I find a new dresser frequently interposing a piece of lint, with or without simple ointment, between the plaster and the sore. It is a very injurious practice; it keeps the sore slopped with its own discharge; it prevents the plaster from making that uniform and regular pressure which is required. When the sore has been healed, the patient should continue to wear the plaster for *some time* afterwards, otherwise the cicatrix will give way, and for the same reason he should *ever* afterwards wear the bandage.

Other methods of treating patients laboring under varicose veins have been proposed by surgeons in former times, and also of late years. They have proposed to relieve or cure the disease by performing operations upon the affected veins. I need not carry you back to the propositions of Celsus on this subject, nor even to those of Heister. I shall only speak to you of methods that have been suggested within the last 30 or 40 years.

Sir Everard Home recommended the application of a ligature, where the veins of the leg were varicose, to the vena saphena major. He performed this operation in a great number of cases, and in a few cases he applied it to the vena saphena minor. When I was a student, nothing was more common than to see a patient with varicose veins standing on a table, and leaning over the back of a chair, to have this operation performed. The skin was divided, a silver needle, armed with a ligature, was passed under the vein, and the vein was tied. In many instances, at first, no ill consequences ensued; but by and by a private patient of Sir Everard Home became affected with venous inflammation, and died. The same thing then occurred in another patient. When I was house-surgeon here, there were two women on whom the operation was performed, in each of whom venous inflammation, attended by typhoid symptoms, supervened. Fortunately they did not die, but they had a very narrow escape. The operation was performed by other surgeons, and *in* their hands also it was found that every now and then venous inflammation was brought on, which ended fatally. The operation was then generally abandoned. Mr. Abernethy remarked,—"I dare say it is only the ligature that brings on the inflammation. You divide veins when you amputate, and they do not become inflamed; why should you not merely cut across the vena saphena, and put on pressure?" He was mistaken in his view of the matter, which was not indeed much understood by surgeons at that time. We now know that the veins, after amputation, not unfrequently inflame, and that this is one of the most common causes of death after amputation. When I was first assistant-surgeon, there was a man with very bad varicose veins; such a case as those in which the vena saphena would formerly have been tied. I did not tie the vein, however, but I followed Mr. Abernethy's advice, cutting it across, and applying a compress and bandage. The patient had venous inflammation, attended with very severe typhoid symptoms, and died within four days after the operation. Since then, as you may suppose, no operation has been performed on the vena saphena, either by ligature or in any other way. There are no circumstances here to justify the performance of a dangerous operation. You may perform dangerous operations to get rid of a disease still more dangerous, but you have no right to perform an operation attended with such a degree of danger as can be appreciated, in order to get rid of a disease which is not dangerous; and no one can say that varicose veins belong to the class of dangerous diseases. But still there is another reason against having recourse to this operation. I do not believe, from anything that I have formerly seen, that the operation permanently benefited the patients. It is true that they appeared to go away a great deal better, but I now and then saw one of them a year or two afterwards, and I always found them as bad as ever. Indeed I am by no means certain that the benefit which the patient appeared to derive, in the first instance, was the result of the operation: and I am more inclined to believe that it arose from his having been necessarily kept for some time in bed in the horrizontal posture. Patients always appear to get better under these circumstances. But I may observe further, that there appears to be no reason why in ordinary cases of varicose veins the obliteration of the saphena major should do any good, and that there are better grounds for believing that it will do harm. If you stop the vena saphena major you prevent the due return of blood to the heart, so that it is likely that the veins will become worse than before. Have I not shewn to you that pressure on large venous trunks causes an obstruction of the blood in passing through them? that this is one common cause of varicose veins? In *very bad cases*, however, of this disease, I can understand why the patient should derive benefit from trying the vena saphena major; and in order that you should understand what I now state, I must explain to you the different cendition of the parts where the veins are very much dilated, and where the disease has only proceeded to a limited extent.

If the veins are but little dilated, or dilated only in particular places, the valves can still continue to answer the purpose for which they are designed. If the vena saphena major be not at all dilated, while the smaller veins of the leg are dilated, the valves of the vena saphena major act perfectly, and take off the weight of the column of blood pressing on the veins below; but if the vena saphena major be itself considerably dilated, its valves then are of no use. I have sometimes seen a very curious result from this. I had a patient, for example, in whom there was an unusually large cluster of varicose veins on the inside of the leg, while the vena saphena major was of enormous diameter, so that the valves could evidently be of no use. If I put on a bandage, and squeezed the blood out of the veins below, and then put my thumb on the vena saphena major above, so as to stop the circulation through it, I found, on taking off the bandage, the patient being in the erect posture, that the cluster of veins below filled very slowly from the capillary vessels. But if the patient, being in the erect posture, I took off my thumb from the vena saphena major, the valves being of no use, the blood seemed to flow down from the trunk of the vena saphena major, contrary to the circulation, and filled the varicose cluster below almost instantaneously. I can understand that a ligature upon the vena saphena major, under these circumstances, would in a great degree lessen the inconvenience arising from the distension of varicose veins below. It would answer the same purpose as the pressure of my thumb; but still it is not to be supposed, that the good thus obtained would counterbalance the chance of mischief resulting from the operation.

I was occupied, many years ago, in making experiments on the obliteration, not of the vena saphena, but of the veins themselves. I applied caustic so as to penetrate through the skin to the veins, and in this way I cured many varicose ulcers. Mr. Mayo has, as I have been informed, employed the same practice lately, with this difference: he has not gone far enough to make a slough of the vein, but brought on some inflammation, which has caused the vein to become obliterated. I tried this method in many cases, but I cannot say that I found it answer sufficiently to make it worth the patient's while to submit to it. The application of the caustic was very painful, the slough took a long time to separate, the sore took a considerable time to heal, and though one cluster was cured, other clusters appeared. Altogether it was a very tedious process, and my own experience does not lead me to recommend it.

Then I contrived another method. Though there is danger in cutting across large veins, or in tying them, there does not appear to be any danger which can be appreciated from the ligature of smaller veins. Piles are nothing originally but varicose veins; now I have performed operations for internal piles, I cannot tell you how often, for there is nothing in the practice of surgery more common; but I have never yet seen a patient have venous inflammation arising in consequence.

We frequently cut across small veins in operations, and they are divided by accident; but we never find venous inflammation supervening. Although there may be danger from operations on the vena saphena major, we have no right to expect danger from operations on the smaller veins. I contrived, then, the following method. Supposing that I intend to cure a particular cluster of veins, I use a sharp pointed bistoury, which cuts, not like a common bistoury, on the concave, but on the convex edge. I puncture the skin with this instrument on one side of the varicose cluster; I carry the blade under the skin, between it and the varicose veins, over to the other side of the cluster; and having carefully performed this part of the operation, the skin over it remaining entire, except where the first puncture was made, I turn the edge of the instrument backwards, and drawing it out, cut across the cluster. A good deal of hæmorrhage follows, but the pressure of a compress commands it, and a bandage is applied afterwards. The wound, in most instances, heals by the first intention. The varicose veins are obliterated, and usually in a few days the patient suffers no inconvenience from the operation. However, in some cases, the wound suppurates, instead of healing by the first intention, which protracts the cure. Then, in other cases, a remarkable occurrence took place. Although I was satisfied that the cluster was divided, the disease was not cured. It seemed as if the veins healed without being closed. As the ductus choledochus, or the intestinal canal, will heal after the application of a ligature, without the continuity of the canal being destroyed, so it appeared that the continuity of the canal of the veins was not in every instance obliterated.

This was a very easy and very safe method of curing varicose veins, yet we hardly ever perform this operation now; for, with my present stock of experience, it really seems to me that there are very few cases in which it is worth the patient's while to submit to it. I have always observed that if I cured one cluster, two smaller ones have appeared, one on each side, so that ultimately I left the patient no better than I found him.

The operation, however, is proper where there is a varicose cluster much distended, and liable to burst and bleed. Here you may actually save the patient's life by having recourse to it; and you may do so without considering whether fresh clusters are or are not likely to form afterwards. Sometimes, when there is a varicose cluster above and below on which a varicose ulcer depends, you get the ulcer to heal sooner than it otherwise would by dividing the cluster. I do not recommend this generally in cases of varicose ulcer, but only every now and then where there is unusual difficulty in getting it to heal. I generally observe that it heals sooner if you divide the cluster below than the cluster above. Then there are some cases where a varicose cluster is productive of an unusual quantity of pain, apparently in consequence of there being some nervous filament lying over it which is kept on the stretch. There you may relieve the patient from the pain of the particular cluster by the division of it. But these occasions are of rare occurrence; and under other circumstances I really do not think that it is worth the while of any patient to submit to the operation.

I ought not to take leave of the subject which is before us, without referring to a very ingenius method of obliterating varicose veins, which has been lately adopted by M. Velpeau, of Paris. He introduces a pin or needle thro' the skin, which is passed underneath the vein, and at right angles to it. A twisted suture is then applied round the two ends of the pin, so as to compress the vein sufficiently to produce its obliteration. I cannot, from my own experience of this practice, say any thing of its advantages or disadvantages; but must acknowledge that it seems not improbable that it may be preferable to the other methods of which I have given you a description. Still, the observations which I have made as to these other methods, apply equally to this. It may be useful in certain cases, and under peculiar circumstances; but I can see no reason to believe that you would be justified in having recourse to it on ordinary occasions."

